# A clinically integrated curriculum in Evidence-based Medicine for just-in-time learning through on-the-job training: The EU-EBM project

**DOI:** 10.1186/1472-6920-7-46

**Published:** 2007-11-27

**Authors:** Sjors FPJ Coppus, Jose I Emparanza, Julie Hadley, Regina Kulier, Susanne Weinbrenner, Theodoros N Arvanitis, Amanda Burls, Juan B Cabello, Tamas Decsi, Andrea R Horvath, Marcin Kaczor, Gianni Zanrei, Karin Pierer, Katarzyna Stawiarz, Regina Kunz, Ben WJ Mol, Khalid S Khan

**Affiliations:** 1 Academic Medical Center, University of Amsterdam, Department of Obstetrics and Gynaecology, Meibergdreef 9, 1105 AZ Amsterdam, The Netherlands; 2 Academic Medical Center, University of Amsterdam, Department of Clinical Epidemiology and Biostatistics, Meibergdreef 9, 1105 AZ Amsterdam, Amsterdam, The Netherlands; 3 CASPe (CASP Espana), Joaquin Orozco 6, 1°-F, 03006 Alicante, Spain; 4 Birmingham Women's Hospital, Metchley Park Road, Edgbaston, Birmingham B15 2TG, UK; 5 The University of Birmingham, Edgbaston, Birmingham B15 2TG, UK; 6 Agency for Quality in Medicine, Weglelystrasse 3, 10623 Berlin, Germany; 7 University of Pécs, Department of Paediatrics, József Attila u. 7, Pécs, H-7623, Hungary; 8 TUDOR, University of Szeged, Albert Szent-Gyorgyi Medical and Pharmacological Centre, Somogyi Bela ter 1, Szeged, H-6725, Hungary; 9 CASPolska, 30-347 Krakow, ul. Wadowicka 3, Poland; 10 Universitá Cattolica del Sacro Cuore, Via Emilia Parmense 84, 29100 Piacenza, Italy; 11 Basel Institute for Clinical Epidemiology, Hebelstrasse 10, CH 4031 Basel, Switzerland

## Abstract

**Background:**

Over the last years key stake holders in the healthcare sector have increasingly recognised evidence based medicine (EBM) as a means to improving the quality of healthcare. However, there is considerable uncertainty about the best way to disseminate basic knowledge of EBM. As a result, huge variation in EBM educational provision, setting, duration, intensity, content, and teaching methodology exists across Europe and worldwide. Most courses for health care professionals are delivered outside the work context ('stand alone') and lack adaptation to the specific needs for EBM at the learners' workplace. Courses with modern 'adaptive' EBM teaching that employ principles of effective continuing education might fill that gap. We aimed to develop a course for post-graduate education which is clinically integrated and allows maximum flexibility for teachers and learners.

**Methods:**

A group of experienced EBM teachers, clinical epidemiologists, clinicians and educationalists from institutions from eight European countries participated. We used an established methodology of curriculum development to design a clinically integrated EBM course with substantial components of e-learning. An independent European steering committee provided input into the process.

**Results:**

We defined explicit learning objectives about knowledge, skills, attitudes and behaviour for the five steps of EBM. A handbook guides facilitator and learner through five modules with clinical and e-learning components. Focussed activities and targeted assignments round off the learning process, after which each module is formally assessed.

**Conclusion:**

The course is learner-centred, problem-based, integrated with activities in the workplace and flexible. When successfully implemented, the course is designed to provide just-in-time learning through on-the-job-training, with the potential for teaching and learning to directly impact on practice.

## Background

There is an increasing imbalance between the exponential growth of medical knowledge and the opportunities available to average clinicians to read, assimilate and apply this information to improve health care. Estimates suggest that 27 kg of guidelines, 3,000 new papers, 1,000 new indexed Medline articles, and 50 new randomised clinical trials (RCTs) are published each day. However, clinicians only manage to spend one hour a week to digest this huge amount of information [1]. Skills to manage knowledge translation efficiently and to distinguish useful evidence from less useful information have become of paramount importance [2]. Teaching programs in evidence based medicine (EBM) face the challenge to impact on knowledge management and transfer skills among health care workers in order to employ the best available evidence in clinical decisions.

EBM is the integration of up-to-date patient-oriented valid research into clinical decision making by doctors and patients. "*Good doctors use individual clinical expertise, the best available external evidence as well as patient preferences, and neither alone is enough. Without current best evidence, practice risks becoming rapidly out of date, to the detriment of patients*[3]." For EBM to be practiced conscientiously, professional qualifications system within health care need to integrate the concepts of problem based learning [4] and lifelong learning [5]. At present, this objective is not yet well achieved in many countries.

Currently, EBM is mainly taught through courses, conferences, workshops, journal clubs or educational meetings, so called "stand alone" courses that are insufficiently integrated into daily clinical practice and clinical postgraduate training [6]. Thereby, physicians go without important opportunities to incorporate the day-to-day problems of real patients in their learning of evidence-based practice. A recent systematic review showed that "stand alone" education improved basic knowledge of EBM, but evidence for improving practice was lacking [7]. For successful implementation in practice, EBM knowledge would need to result in skills, attitudes and appropriate changes in behaviour [8-11]. An improvement on each of these four domains can best be achieved, when courses are integrated into daily clinical routine practice leading to just-in-time learning through on-the-job training [7,12].

Increasingly, it is required that health care decisions are based on sound evidence [13]. Experts agree that EBM should be a mandatory skill for postgraduate training and continuing medical education [2]. However, EBM remains a difficult-to-teach subject and a joint agreed educational approach within many European countries and certainly across Europe is lacking. In 2005 the Leonardo da Vinci Community Vocational Training Action Programme for life long learning, funded by the European Union, provided a grant for a pilot project to fill this gap in medical professional development.

We report on this initiative named the European Union (EU)-EBM project that aspires to develop a clinically integrated EBM course. In a pilot project, we designed and developed the first teaching unit, with emphasis to its promotion and piloting across the European health care sector and beyond. This paper describes the process of curriculum development along with its current results.

## Methods

The goal of the curriculum is to develop a postgraduate course integrated into daily clinical practice allowing a maximum of flexibility to learners.

A curriculum committee consisting of experts in the field of EBM, clinical epidemiologists, clinicians and educationalists from the participating countries commenced the work. We based the design and development of the EBM curriculum on an educationally sound methodology [14] including the following domains:

• identification of EBM needs in each partner country

• formulation of the aims, objectives and learning outcomes of the curriculum

• development and organisation of the content of the curriculum

• development of the teaching methods

• definition of the educational strategy and educational environment

• definition of the assessment strategy

• communication of the curriculum to learners

• the overall management of the process.

In addition, an independent European Steering Committee provided input into the content, educational approach, applicability and sustainability, giving advice at critical stages in the curriculum development and thereby assured external validation and triangulation of the syllabus prepared.

## Results

### Identification of EBM needs

First we identified specific needs of learners, which could direct the curriculum development. Definitions needed to take into account differences in levels of EBM education and the variety of teaching and learning opportunities across European health care systems. This was mainly done by an in-depth discussion among the participating experts at the beginning of the project, who had a good overview of the ongoing activities in their countries, with input from the external steering committee. This process helped to identify definable educational goals for the documented learning needs. During meetings, consensus was achieved in a Delphic fashion [15] on the need for an initial course dealing with systematic reviews of the effectiveness of interventions, and with teaching tuned to the basics of EBM to make a standardised introduction to practicable EBM.

### The aims, objectives and learning outcomes

The aim was to familiarise course participants with EBM basics to help them incorporate evidence from systematic reviews on therapeutic interventions into daily clinical practice. It was stipulated that the course should be clinically integrated with a large component of e-learning. To meet these objectives we aimed at short individual sessions to allow learning at the worksite during a short break, with the e-learning modules structured in a way they can be easily interrupted and taken up again. The following learning objectives and outcomes were identified: "After completion of the course, participants should first be able to generate structured questions arising from problems in their own clinical practice in PICO (population, intervention, comparator and outcome) format [16]. Although other formats exists [17-19] we felt that this was the one most well-known and most easy to understand design for a course tuned to basic EBM teaching. Secondly, they should be able to search for relevant literature, aiming for and identifying systematic reviews wherever possible. Third, they should be able to assess the quality (validity) of systematic reviews and the primary research included within them. Fourth, they should be able to assess the applicability and generalisability of the research findings to their own clinical practice. Finally, they should demonstrate their understanding of strategies to effectively implement new research findings into clinical practice, including knowledge of barriers that often block such implementation in clinical practice [1,20]." These key issues guided the subsequent steps in curricular development.

### The curricular content

The curriculum is subdivided into five modules each of which addresses core competencies in evidence-based practice.

These modules are:

1. Asking and framing clinical questions.

2. Searching for the evidence

3a. Critical appraisal of primary randomised controlled trials

3b. Measures of effectiveness

3c. Critical appraisal of systematic reviews of intervention studies

4. Applicability of the evidence to the patient

5. Implementation of the evidence into practice

### Organisation of the content and teaching methods

At the beginning of the course, learners and facilitators receive a handbook with the overall aims and objectives of the curriculum and modules, relevant additional methodological and clinical papers, an outline of the teaching, learning and assessment strategy, and its timetable. The 5 modules of the course are delivered sequentially over a period of 5 weeks. Order and sequence of the teaching sessions ensure that the prerequisites and the basic content appear first while more advanced content appears later. We refer to Table 1 for a comprehensive overview of the curriculum.

**Table 1:** An overview of EU EBM curriculum

**Aim: **To familiarise course participants with evidence based medicine (EBM) basics to help incorporate evidence from systematic reviews into practice.

**Target participants: **Health professionals in a clinical setting.
**Learning objectives:**
Upon the completion of the course, participants should be competently able to:
• generate structured questions arising from clinical problems in practice
• search relevant literature, identifying systematic reviews wherever possible
• assess the quality (validity) of systematic reviews and primary research included within them
• assess the applicability of research findings in clinical practice
• effectively implement the output from above activities into clinical practice

**Reading/Learning Resource:**
• A study guide outlining the course and providing learning exercises/assignments.
• E-learning modules
Five models provide learning materials for undertaking the exercises in the study guide
Module 1: Asking clinical questions
Module 2: Searching the evidence
Module 3: Critical appraisal of systematic reviews
Module 4: Applicability of the evidence to the patient
Module 5: Implementation of evidence into practice

**Learning/teaching methods**
• Participant initiated (tutor facilitated) small group work and one-to-one teaching and learning in a clinical setting
• Clinical tutor will guide participants in a clinical setting:
◦ Identifying learning opportunities in a clinical setting
◦ Directing appropriate use of learning resources
◦ Providing feedback on learning exercises/assignments
• Participants will pursue independent study using the study guide and e-learning modules directed/facilitated by the clinical tutor and will undertake summative assessments

**Assessments**
• Formative
◦ Feedback on assignments recorded in the study guide
• Summative
◦ Multiple choice questions to test knowledge
◦ Questionnaire to test attitudes

**Figure 1:**
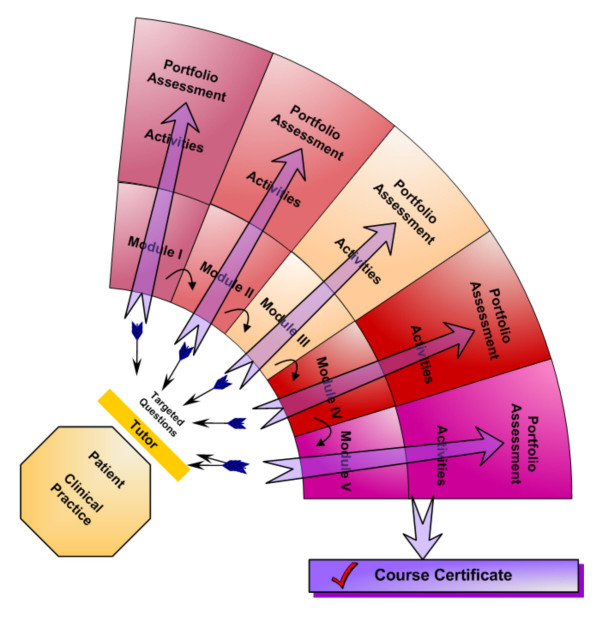
**An overview of teaching and learning activities in the EU EBM course**. Graphical representation of the EU-EBM curriculum. A facilitator picks up problems encountered with patients in daily clinical work and helps in formulating targeted questions. Hereafter, the learner attends the e-learning modules and consequently completes small activities and an assignment. All five modules are collected in a portfolio. After successful completion of the course, the student will receive a course certificate.

**Figure 2:**
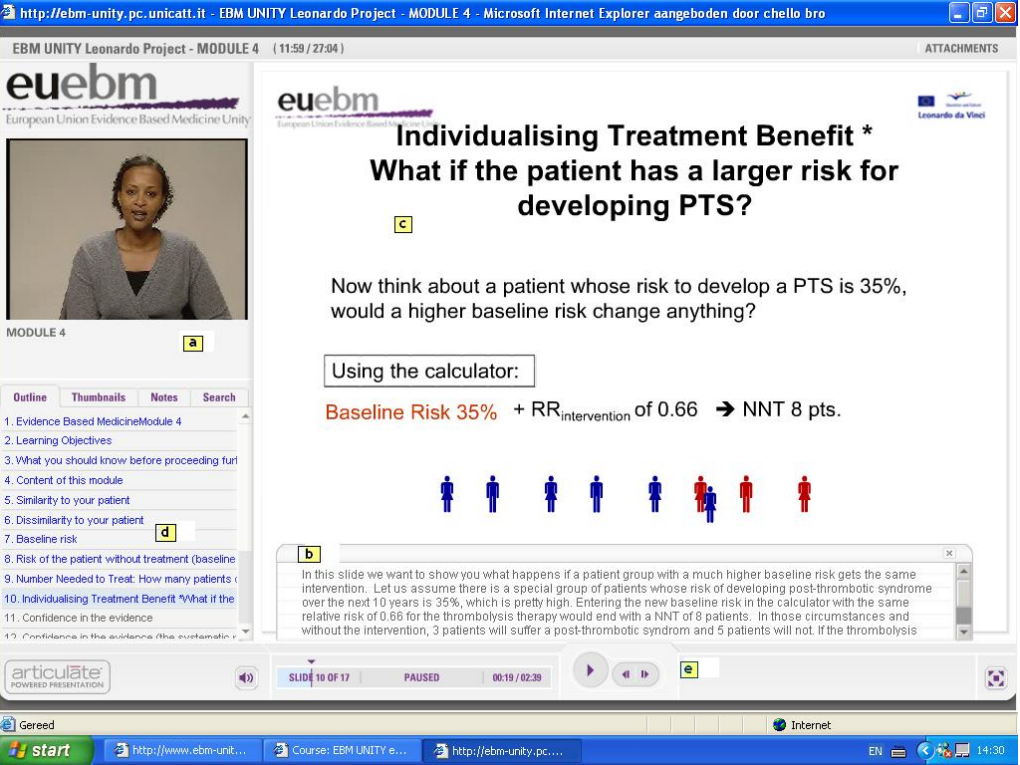
**Screenshot from one of the EU-EBM e-learning modules**. Screenshot showing the various audiovisual teaching modalities that are applied in the e-learning session to support self-directed learning: a) a person is talking to the learner; b) the learner can follow the presentation by reading the notes; c) the slide summarizes the core content of the presentation; it may contain hyperlinks to other topics in the same module; d) the sidebar provides orientation to the learner about the content of the lecture; e) the bottom bar allows the learner to pause, or quickly navigate forth and back.

Each of the sessions integrates a combination of teaching methods: (Figure 1):

• A facilitator (e.g. the supervising consultant) plays a central role in the clinically integrated curriculum by acting as a moderator in the learning process of the learner, by identifying learning opportunities encountered by the learners in daily care for patients and by directing appropriate use of learning resources in a clinical setting. This could be done during ward-rounds, daily meetings when shifts are transferred, in fact the facilitator can point out learning opportunities in every patient-doctor encounter.

• Self-directed, independent e-learning via the Internet or CD-ROM. These sessions (one or more per module) are structured such that a learner can work them through in 10 to 20 minutes. This allows maximum flexibility to the learner and easy access from multiple sites, both inside and outside the hospital, which addresses the needs of a busy clinician. The e-learning materials consist of slides and written scripts; a talking head which covers the content of the scripts and guides the lecture; play, pause and skip options; and hyperlinks to main sections within the sessions. An example of one of the e-learning modules is shown in Figure 2. Sessions can be accessed multiple times if necessary. These e-learning sessions aim to provide basic understanding of the key concepts covered in each module.

• A variety of minor activities where learners perform practical tasks and thereby transfer their knowledge into skills. The learner is expected to frame five clinical questions according to the PICO format from problems encountered in daily practice, to develop two structured search strategies in Pubmed, to appraise a randomised clinical trial on the topic on several validity items, to identify the (dis)similarity of their clinical patient with the patients included in the systematic review, and to read an essay on barriers that exist when implementing EBM in daily clinical practice.

• Each module ends with an assignment that learners hand in to the facilitator, such as the search used to identify a systematic review for one of PICO questions formulated in the first module, or a paper on the learner's deliberations on how to implement the found evidence in their own clinical setting. By providing feedback on learning exercises/assignments, these assignments can act as a starting point for a one-to-one interaction between learner and facilitator or a small group discussion in the resident's regular teaching session. Before moving to the next module, learners will finish each module with a multiple choice questionnaire that covers the content of that specific module. All completed assignments contribute to the learner's documented portfolio.

### Educational strategy and educational environment

**Figure 3:**
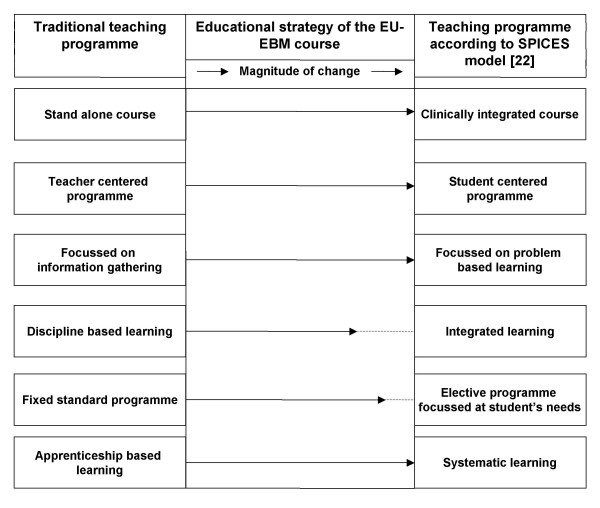
**An overview of educational strategy of the EU-EBM curriculum according to the SPICES model**. The educational strategy of the EU-EBM curriculum according to the SPICES model [19] moving away from traditionally, teacher centred educational programs to student orientated, problem based learning programs. The size of the arrows in this figure indicates the magnitude of change between a traditional and the EU-EBM course.

Many educational models have been developed on which teaching strategies can be based [21]. We chose to incorporate our EBM curriculum within the SPICES framework, in which six educational strategies are represented as a gradual continuum, contrasting the more innovative SPICES model with more traditional teaching strategies [22]. As the educational environment influences participants' motivation, learning and thinking, the curriculum aims to shift from a teacher-centred orientation to a participant-centred approach [14,22]. We aim for an ethos in which facilitators and participants regard each other as professional colleagues for whom teaching and learning works as a two-ways process. A carefully designed learner's handbook guides participants through the curriculum systematically and the e-learning material allowing learners to advance at their own time, speed and needs and to organise the learning easily around their clinical responsibilities. Appraising the applicability of the evidence in their own setting to their own patients supports the clinical focus of learning in a problem-based fashion. Although our curriculum contains fixed learning objectives that have been precisely matched against the learning materials, for most modules elective course material is provided. Whether or not learners will access additional materials will be largely driven by students' intrinsic motivation and specific needs. The full educational strategy is summarised in Figure 3. As part of the pilot project, the impact of our curriculum and its implementation in the educational environment is studied by using an adapted version of the DREEM questionnaire [23].

### Assessment strategy

The course will be piloted formally on ten doctors per participating country (in total over 70 doctors). The curriculum assessment concerns evaluation of participants' knowledge, skills and attitudes through formative and summative assessments. For this, a multiple choice questionnaire (MCQ) was modelled according to the validated Berlin Questionnaire [24] that evaluates the learner's knowledge and skills by following closely the learning objectives of each module. In addition to this, as part of the pilot phase, an existing validated tool for attitudes towards EBM [25] -slightly adapted for our purpose- will assess learners' attitudes towards the clinically integrated e-learning course and EBM in general. Pre-course attitudes will be compared with the learners' attitudes after completion of the course. In addition, during the pilot phase, we will collect participants' and facilitators' feedback on the course by using semi-structured qualitative interviews, the results of which are used to identify lack of clarity or weaknesses in course material. Information of the level of difficulty, the time and preparation spend by both learners and facilitator is collected, and the level of comfort of facilitators with facilitating the course is explored. All these results will help us to refine the curriculum and to match learner's needs even more precisely.

### Management of the process

To assure sustainability, an independent steering committee of clinical epidemiologists, EBM experts and educational experts from countries both participating and non-participating in the partnership overlooked the development of the curriculum, content of the teaching materials, its assessment and the evaluation of the pilot project. The committee gave advice to strategic questions addressing implementation and dissemination across Europe and provided external feedback to the group.

On national levels the project aims to obtain certification by continuous medical education agencies, in line with the Copenhagen Declaration (2002) of the European Union [26], which is currently preparing a European Credit (Transfer) System for Vocational Education and Training (ECVET). ECVET aims to facilitate mobility in vocational education and training, allowing young people to transfer learning results from one country to another in concordance with national regulations [27].

## Discussion

The EU-EBM curriculum presented in this paper targets postgraduate training and continuing education, using a clinically integrated e-learning methodology. To our knowledge, clinically integrated teaching of EBM is currently not provided in Europe. Furthermore, existing courses do not have a comparable format with continuous repetitive learning over a longer period, which includes small activities and individualised assignments to acquire skills and to deepen knowledge, standardised assessments at the end of each module and special emphasis on implementation of evidence in practice. Furthermore, one of the main innovations here is to get European countries to work together in order to harmonize an integrated teaching of EBM, which has not been subject of any harmonization effort before.

The main purpose of the curriculum is to provide doctors with the basic skills needed to practice and implement evidence-based therapy. For this, the course covers the 5 key steps of evidence based practice, namely framing the clinical question, searching for the evidence, appraisal and interpretation of the evidence, applying the evidence to the patient and implementing the evidence into practice, using problems encountered in daily patient care. An e-learning platform [28] or access through CD-Rom ensures timely and immediate access to the necessary methodological knowledge and background materials. The development, piloting and certification of a basic clinically integrated course in EBM offers the learner the opportunity for a standardised acquisition of EBM skills, promotes life long learning, and increases the mobility of doctors within a unified region such as the European Union.

A distinct feature of the EU-EBM curriculum is the low threshold approach to facilitate integrated EBM teaching and thereby addressing specific problems of the target group. Teaching basic concepts in short learning units and allowing maximum flexibility in the location of the learning takes into account the timeframe and working patterns of busy clinicians. The course separates the practice of EBM in the day-to-day patient encounter (framing questions from patients' problems, identifying the literature and applying the evidence to the individual patient) from the acquisition of methodological knowledge (critical appraisal of clinical research) which is mainly taught on the e-learning platform. An EBM enthusiastic senior clinician can act as a moderator in this learning process, but is not expected to teach the methodology of EBM. This division of responsibilities fosters the spread of EBM-teaching and learning to clinical settings with strong interest for EBM but with a lack of specialists teaching the methodology of critical appraisal. Inspired learners will receive additional reading material and guidance on how to advance their skills. Furthermore, groups of residents could pass the course simultaneously as part of their in-house training. We envision that this joint learning experience might stimulate constructive critical reflection on current clinical practice beyond the specific course content, initiating a new culture of discussion and EBM-practice within a department or unit. Last but not least, the translation of the curriculum in French, German, Spanish, Hungarian and Polish should help non-native English speakers to learn new concepts in their own language and ensure greater accessibility of the learning materials.

The key aims of this project will need to be met by raising awareness of the importance of training in EBM throughout Europe as a means of enabling health care professionals to provide better care to patients [13] and ultimately encouraging the inclusion of the qualification developed into mainstream education. The dissemination systems in place such as the website [28], presentations on national conferences and workshops, word of mouth and publications in national language will allow access, discussion and dissemination of the results. The project partners started already to share the knowledge and experience from this project with their national networks of institutions that might benefit from the process. Many organisations and institutions look for "easy to use" learning programs in EBM that they can integrate -with limited resources- in their local working environment. We further encourage the incorporation of this course in existing educational lessons offered by other institutions, which will then hopefully form the established network for an exchange of training practices and experiences. Dissemination to good educational practice when successful can help to create a level playing field for mobility of doctors and other clinical professions.

So far, in this pilot project, we only covered one unit of EBM, namely systematic review of therapeutic effectiveness, due to time and financial constraints. Furthermore, the current program is designed for medical doctors. However, numerous organisations of allied health professionals already expressed interest in this course as did consumer organisations and patient self-help groups. We need testing of the existing course in those groups and modify it according to their learning needs. Furthermore, the course is so far only available in six different languages. Additional translation in other European and non-European languages might be warranted. The curriculum assessment currently contains evaluation of participants' knowledge, skills, and attitudes. Although we acknowledge that behavioural changes are essential to fully implement life long learning, measurement of such changes require longer follow-up than is possible within the current duration of this pilot-project.

Once fully implemented, the ultimate outcome of this pilot project will be a European qualification in EBM, which will be used by doctors, hospitals, professional bodies responsible for postgraduate qualifications and continuous medical education. In the long term this project has the potential to benefit the general public as EBM will contribute to a more transparent healthcare system, better-informed patients and a generally better informed society, which is what EBM is all about.

## Conclusion

The EU-EBM project has shown that it is possible to harmonize EBM teaching across Europe, with the development of a clinically integrated course based on e-learning technologies. The course is designed to provide just-in-time learning through on-the-job-training, with the potential for teaching and learning to directly impact on practice.

## Competing interests

The author(s) declare that they have no competing interests.

## Authors' contributions

SC, JE, JH, BWM, RMK, RAK and KSK drafted the first version of the manuscript. All authors revised the manuscript and approved final submission for publication. All authors are member of the EU-EBM and were involved in the design, development and implementation of the course described.

## Pre-publication history

The pre-publication history for this paper can be accessed here:


